# Supplementary material for: Sequential islet transplants for type 1 diabetes are not associated with sustained or cumulative increases in hepatic portal venous pressure
Source: PLoS One. 2025 Jul 29;20(7):e0329074. doi: 10.1371/journal.pone.0329074 (PMC12306781; doi:10.1371/journal.pone.0329074)
Supplement: Attachment — Submitted filename: Response to Reviewers.docx [file pone.0329074.s001.docx]

April 01, 2025

Alice Carr

1-004 Li Ka Shing Centre for Health Research Innovation,
Edmonton, 
Alberta, Canada T6G 2E1

Dear Dr Rafiqi,

We are pleased to resubmit our manuscript entitled “Sequential Islet Transplants for Type 1 Diabetes Are Not Associated with Sustained or Cumulative Increases in Hepatic Portal Venous Pressure”.

We thank the reviewers for their time and comments, and have made effort to address them in full. Point-by-point responses are in **bold** below. Changes to the text are highlighted in yellow within the “Revised Manuscript with Track Changes”. In addition, we provide the information required to meet the journal requirements as requested by the editor.

We would like to highlight a slight change to Table 1, as we noticed that the incorrect proportions were printed for intra-portal infusion counts. We have updated this, highlighting the changes within the table itself and have also changed the text where described on lines 259-263. Additional changes as requested by the reviewers or editor are detailed throughout the point-by-point response below.

Further, as requested, we would like to confirm that our competing interest statement should include the additional statement: *This does not alter our adherence to PLOS ONE policies on sharing data and materials.*

Additionally, we would like to highlight the additions to our ethics statement, as appropriate, which now reads as below:

*This study was approved by our institutional (University of Alberta Hospital) health research ethics board (PRO000001120). Written informed consent for the use of health data for research purposes was obtained from all participants. The informed consent and organ donation processes are subject to the Government of Alberta’s Human Tissue Organ and Donation Act or local Provincial Equivalent which requires donor anonymity. Consent may be withdrawn at any point during the donation process. Organ donation is considered as a gift and neither the donor nor consenter receive any benefit. Information from donor health records are subject to provincial law and policy and as such public access to health information must be requested through a formal process. The full act can be found here:* [*https://open.alberta.ca/publications/h14p5*](https://open.alberta.ca/publications/h14p5)*.Minors may have been a part of this donation cohort. All consent is performed under specific policy requirements that protect vulnerable populations and outline who may give informed consent. The basic principles include: Requires capacity (or alternate decision maker), Must be Informed, Must be Specific, Must be Voluntary, Requires Understanding, Must be Documented. The full policy statement can be found here:* [*https://extranet.ahsnet.ca/teams/policydocuments/1/clp-consent-to-treatment-prr-01-05-procedure.pdf*](https://extranet.ahsnet.ca/teams/policydocuments/1/clp-consent-to-treatment-prr-01-05-procedure.pdf) *”*

We have ensured the information in the ethics statement is reflected in the Methods section of the manuscript and highlighted throughout the response below, the lines at which these changes occur.

We thank you for taking the time to consider our resubmission of this manuscript,

On behalf of all co-authors, yours sincerely,

**Dr Alice Carr, PhD**

**Review Comments to the Author**

Reviewer #1: Overall a well written manuscript with a clear and concise message. The cohort studied is the largest and most significant cohort worldwide and therefore the study is of particular significance. The authors make the point clearly that this is of relevance and translational to the field in view of the stem cell islet transplantations that are now happening. It is therefore a useful addition to the literature.

**We thank the reviewer for their kind comments regarding this manuscript. We address the minor suggestions as below:**

1. line 156 - a little ambiguous – reword

**Thank you for this suggestion. We have reworded as below:**

**Original: Procedures were not initiated or paused during infusion if an elevated pressure > 20mmHg was observed.**

**Updated lines 192-193: The procedure was not initiated if an initial pressure > 20 mmHg was observed, or were paused and assessed if pressures reached > 20 mmHg during the infusion**

2. Line 164 - I am not certain what is meant by the term random intercept

**Thank you for highlighting this. Mixed effects models with a random intercept were used to accommodate individual-level differences in the response variable, portal pressure change, across repeated measures. This approach allows us to analyze how specific parameters of the islet cell preparation (PCV, purity, IEs) and SLV relate to changes in portal pressure, while accounting for inherent variability between individuals over time.**

**To make this clearer, we have edited the text as below:**

**Original: Mixed effects models accounting for individual-level differences (random intercept), were used to assess the relationships between change in portal pressure, the specific parameters of the islet cell preparation: PCV, purity and IEs as well as SLV**

**Updated lines 201-205: Mixed effects models with a random intercept, were used to assess the relationships between change in portal pressure, the specific parameters of the islet preparation: PCV, purity and IEs as well as SLV, with the random intercept accommodating individual-level differences in the response variable, portal pressure change, across repeated measures.**

**We have also removed the additional occurrence of “(random intercept)” from line 198.**

3. Table 3 - on my version the portal pressure values are not displayed clearly.

**We ensured on first round to upload high DPI figures or .docx files for tables. Quality perhaps was reduced on compilation for reviewers. We will communicate with the editor to ensure the figures and tables remain of high quality in the final document.**

4. In the discussion I would solely discuss the results. Please take out Figure 4 and 5 and place these and describe as necessary in the results section. These can then be discussed in the discussion

**Thank you for this suggestion. We have taken this onboard and restructured the results and discussion to reflect this.**

**We add to the results section to describe Figure 4 over lines 343-346:**

**Exploratory visualisations of the interaction between PCV and purity, using the final combined model and considering the SLV of an average islet recipient (mean SLV 1524 cm^3^), demonstrated that most procedures are likely to result in acute increases in portal pressure between 2-4.5 mmHg (Fig 4).**

**Which is reflected in our discussion on lines 415-418 beginning with the paragraph:**

**Exploratory visualisations of the interaction between PCV and purity, using the final combined model and considering the SLV of an average islet recipient (mean SLV 1524 cm^3^), demonstrated that most procedures are likely to result in acute increases in portal pressure between 2-4.5 mmHg. Notable from this exploratory visualization…..”**

**We add to the results section to describe Figure 5 over lines 356-362**

**New section heading: The acute portal pressure rise per procedure has remained relatively stable over 24 years of practice in our Centre.**

**Overall the acute portal pressure rise per procedure has remained relatively stable overtime (Fig 5). However, before 2005 there were a higher proportion of procedures resulting in acute portal pressure rises exceeding 4.5 mmHg (38/119 (31.9%) vs. 77/574 (13.4%); Chi-squared 23.1; p<0.0001), and indeed more cases using PCV >5ml (Fig 5) (36/119 (%) vs. 9/574 (%) Chi-squared 128.8; p<0.0001).**

**Which is reflected in our discussion on lines 441-447 beginning with the paragraph:**

**It is noteworthy that over 24 years of practice in our Centre, the acute portal pressure rise per procedure has remained relatively stable overtime. However, as described, before 2005 there were indeed a higher proportion of procedures resulting in acute portal pressure rises exceeding 4.5 mmHg. This coincided with a higher frequency of cases using PCV >5ml occurring also before 2005 – a likely contributing factor to these procedures resulting in acute portal pressure rises exceeding 4.5 mmHg.**

Reviewer #2: The authors previously reported that an increase in portal vein (PV) pressure (PVP) during the ITx procedure compared to pre- and post- treatment levels. Critical complications such as PV thrombus had occurred when the increased poral vein pressure exceeded 4.5mmHg. In this paper, an acute PVP elevation of less than 2 mmHg was investigated at a lower level. Small fluctuations of PVP were reversible, making their evaluation valuable as a safety indicator based on suitable PVC range and calculated SLV adjustment. This paper is valuable for indicating the safety margin during the PV infusion in ITx. However, a few points should be cleared out to understand the detail for ITx method in this journal.

**We thank the reviewer for their kind comments regarding this manuscript. We address the minor comments as below:**

Minor

1. PVC information should be cleared out such as basic medium, cell density, dropping speed, viscosity and whether total volume is fixed or not.

**Our standard approach to assessing packed cell volume is to visually estimate the volume of cellular material in the bottom of 50 mL conical tube following centrifugation at 1100 rpm for 1 minute and rounded to the nearest 0.5 mL. For infusion, islets were suspended in a CMRL based transplant media, with no more than 5cc per 100 ml as described previously. We have added this to the methods as below.**

**Adding to lines 178-181**

**“Our standard approach to assessing PCV is to visually estimate the volume of cellular material in the bottom of 50 mL conical tube following centrifugation at 1100 rpm for 1 minute and rounded to the nearest 0.5 mL..“**

**In addition we have added for clarity within lines 185-190:**

**“The transplant procedure has been described previously [2] Briefly, isolated islets were prepared as previously described [2] by suspension in xenoprotein-free media with no more than 5 cc of PCV per 100 mL of suspension medium mixed with 70 U/kg of heparin (procedures prior to 2005 used 35 U/kg of heparin) and transplanted intra-hepatically via the portal vein accessed by a percutaneous transhepatic approach.[19]”**

2. To evaluate SLV, the liver function including imaged based assessments should be made clear.

**Thank you for this comment. We do not used image-based assessment in the evaluation of Liver Volume and outline the use of the Vauthey et al equation in the methods on line 206. However, we recognise that estimated Total Liver Volume (eTLV) may be a better description of the use of this equation rather than Standard Liver Volume (SLV)- we have hence changed this throughout the manuscript replacing SLV with eTLV, including in the title of Figure 4.**

**We chose this equation as it provides a reliable, population-specific (Western-adults) approximation of liver volume, in the absence of imaging and other parameters, and has shown good correlation (r² = 0.46) with CT-derived liver volumes across multiple centers (Vauthey et al 2002). While it offers a practical and standardized approach for estimating liver volume, we acknowledge the lack of imaging remains a limitation, as individual anatomical variability or underlying liver pathology cannot be accounted for.**

**Journal Requirements**

1. Please ensure that your manuscript meets PLOS ONE's style requirements, including those for file naming. The PLOS ONE style templates can be found at <https://journals.plos.org/plosone/s/file?id=wjVg/PLOSOne_formatting_sample_main_body.pdf> and <https://journals.plos.org/plosone/s/file?id=ba62/PLOSOne_formatting_sample_title_authors_affiliations.pdf>

**We have ensured the manuscript complies with the style requirements outlined.**

2. We note that your study involved tissue/organ transplantation. Please provide the following information regarding tissue/organ donors for transplantation cases analyzed in your study.

- Please provide the source(s) of the transplanted tissue/organs used in the study, including the institution name and a non-identifying description of the donor(s).

**Pancreata from deceased organ donors located in Canada (after neurological or cardiac death) were recovered by Give Life Alberta - Organ Donation North (previously Human Organ Procurement and Exchange program) within Alberta Health Services or the Provincial Organ Donation Organization after informed consent from the donor family has been obtained. The informed consent and organ donation processes are subject to the Government of Alberta’s Human Tissue Organ and Donation Act or local Provincial Equivalent which requires donor anonymity. Information from donor health records are subject to provincial law and policy and as such public access to health information must be requested through a formal process. The full act can be found here:** [**https://open.alberta.ca/publications/h14p5**](https://open.alberta.ca/publications/h14p5)**. We have added this information to our methods lines 133-143 as below and reflected this in our ethics.**

**It is also important to emphasise that the number of people in this study do not directly reflect the number of donors. Islet transplantation within one infusion can include islets isolated from more than one donor and, as described in this manuscript, recipients can often receive more than one infusion over time.**

**Lines 133-143: Pancreata from deceased organ donors located in Canada (after neurological or cardiac death) were recovered by Give Life Alberta - Organ Donation North (previously Human Organ Procurement and Exchange program) within Alberta Health Services or the Provincial Organ Donation Organization after informed consent from the donor family has been obtained. Consent may be withdrawn at any point during the donation process. The informed consent and organ donation processes are subject to the Government of Alberta’s Human Tissue Organ and Donation Act or local Provincial Equivalent which requires donor anonymity. Information from donor health records are subject to provincial law and policy and, as such public access to health information must be requested through a formal process. The full act can be found here:** [**https://open.alberta.ca/publications/h14p5**](https://open.alberta.ca/publications/h14p5)**.**

- Please state in your response letter and ethics statement whether the transplant cases for this study involved any vulnerable populations; for example, tissue/organs from prisoners, subjects with reduced mental capacity due to illness or age, or minors.

**It is possible that minors may have been a part of this donation cohort. It should be highlighted that all consent is performed under specific policy requirements that protect vulnerable populations and outline who may give informed consent. Basic principles include: Requires capacity (or alternate decision maker), Must be Informed, Must be Specific, Must be Voluntary, Requires Understanding, Must be Documented.** **Policy can be found here** [**https://extranet.ahsnet.ca/teams/policydocuments/1/clp-consent-to-treatment-prr-01-05-procedure.pdf**](https://extranet.ahsnet.ca/teams/policydocuments/1/clp-consent-to-treatment-prr-01-05-procedure.pdf)

**Further to this, we would like to highlight that organ donation is considered as a gift and neither the donor nor consenter will receive any benefit. Lastly, consent may be withdrawn at any point during the donation process.**

**We have added to our ethics statement, which is reflected in the Methods section on lines 154-160:**

**Lines 154-160: “Minors may have been a part of this donation cohort. All consent is performed under specific policy requirements that protect vulnerable populations and outline who may give informed consent. The basic principles include: Requires capacity (or alternate decision maker), Must be Informed, Must be Specific, Must be Voluntary, Requires Understanding, Must be Documented. The full policy statement can be found here:** [**https://extranet.ahsnet.ca/teams/policydocuments/1/clp-consent-to-treatment-prr-01-05-procedure.pdf**](https://extranet.ahsnet.ca/teams/policydocuments/1/clp-consent-to-treatment-prr-01-05-procedure.pdf) **”**

- In the Methods, please provide detailed information about the procedure by which informed consent was obtained from organ/tissue donors or their next of kin. In addition, please provide a blank example of the form used to obtain consent from donors, and an English translation if the original is in a different language.

**We have added to our Methods section, on lines 144-153, the below statement:**

**Lines 144-153: Individuals who wish to become organ donors can indicate this by signing (witnessed) their provincial health card, or joining the organ donation registry, but ultimately a family member or next of kin will be required to sign a consent form saying they have been informed about, and agree with, the donation process, even if the person that died has signed the back of his or her Alberta Personal Health Card or registered. Families of deceased individuals who have not indicated in advance their wish to donate an organ may be approached by medical staff to seek family consent. The informed consent process can be accessed: https://myhealth.alberta.ca/alberta/Pages/organ-and-tissue-donation-consent-to-donate.aspx.**

**We have uploaded a blank example form used to obtain consent.**

- Please indicate whether the donors were previously registered as organ donors. If tissues/organs were obtained from deceased donors or cadavers, please provide details as to the donors’ cause(s) of death.

**Some donors may have previously been registered as donors as described above, but not all. We do not know the identity of donors, nor whether they had registered as donors. Causes of death include stroke, hypoxia, overdose and medical assistance in dying.**

- Please provide the participant recruitment dates and the period during which transplant procedures were done (as month and year).

**We believe we have detailed this in the manuscript on lines 113-114:**

**“This is a retrospective study of patients receiving intraportal ITx at the University of Alberta Hospital between May 1999 to April 2023.”**

- Please discuss whether medical costs were covered or other cash payments were provided to the family of the donor. If so, please specify the value of this support (in local currency and equivalent to U.S. dollars).

**As we have outlined above, organ donation is considered as a gift and neither the donor nor consenter receive any benefit.**

- Thank you for stating the following in the Competing Interests section:
  *BAMG is a co-inventor for a patent on TNFRSF25-mediated treatments of immune diseases and disorders (PCT/US2020/053085). PAS has received grants from JDRF, CIHR and Brain Canada and consulting or personal fees from Abbott, Bayer, Dexcom, Eli Lilly, GSK, Insulet, Novo Nordisk, Sanofi, Vertex. He served previously as board chair for Diabetes Canada and is a co-lead for Diabetes Action Canada's innovations in type 1 diabetes goal group. AMJS has received grants or contracts from the Breakthrough T1D (formally Juvenile Diabetes Research Foundation), Canadian Stem Cell Network, Diabetes Research Foundation in Canada, ViaCyte, and the US National Institute of Diabetes and Digestive and Kidney Diseases; serves as a consultant to Vertex Inc, Betalin Inc, and  Aspect Biosystems Inc; and is a co-inventor for a patent on TNFRSF25-mediated treatments of immune diseases and disorders (PCT/US2020/053085) and for a Cellular Transplant Site- Device-less technology (US 14/863541, CA.286512). All other authors declare no competing interests.*

  Please confirm that this does not alter your adherence to all PLOS ONE policies on sharing data and materials, by including the following statement: "This does not alter our adherence to PLOS ONE policies on sharing data and materials.” (as detailed online in our guide for authors <http://journals.plos.org/plosone/s/competing-interests>). If there are restrictions on sharing of data and/or materials, please state these. Please note that we cannot proceed with consideration of your article until this information has been declared.

  Please include your updated Competing Interests statement in your cover letter; we will change the online submission form on your behalf.

**We have added this into the cover letter provided with this point-by-point response, as above.**

- We note that you have indicated that there are restrictions to data sharing for this study. For studies involving human research participant data or other sensitive data, we encourage authors to share de-identified or anonymized data. However, when data cannot be publicly shared for ethical reasons, we allow authors to make their data sets available upon request. For information on unacceptable data access restrictions, please see<http://journals.plos.org/plosone/s/data-availability#loc-unacceptable-data-access-restrictions>.

  Before we proceed with your manuscript, please address the following prompts:
  - If there are ethical or legal restrictions on sharing a de-identified data set, please explain them in detail (e.g., data contain potentially identifying or sensitive patient information, data are owned by a third-party organization, etc.) and who has imposed them (e.g., a Research Ethics Committee or Institutional Review Board, etc.). Please also provide contact information for a data access committee, ethics committee, or other institutional body to which data requests may be sent. Please update your Data Availability statement in the submission form accordingly.

**Our HREB requires that patient confidentiality is maintained. As previously stated, it will not be possible to de-identify the data set sufficiently to avoid risk of identification, particularly those whom have had more than one infusion, due to the lower numbers of participants with more infusions (only 8 individuals with 5 infusions). As an example, by triangulating age, gender and time since transplant it would be easy to identify the individual. As this was an investigation of number of infusions with portal pressure change it is not possible to provide a dataset without this information. The uniqueness of the procedure and known location of the University of Alberta’s Clinical Islet transplant program, create substantial possibility of breaching patient privacy and would breach requirements of our HREB.**

**Our data availability statement therefore reads:**

**Our Human Research Ethics Board requires that patient confidentiality is maintained. Due to the uniqueness of islet transplantation, the relatively low numbers receiving this treatment per year and the known location of the University of Alberta’s Clinical Islet transplant program, there is a risk that the data underlying the results may contain potentially identifying information. Therefore, the data underlying the results reported in this Article are not publicly available. De-identified individual participant data, as well as a data dictionary, can be made available to researchers who provide a methodologically sound proposal with ethics approval. Proposals should be directed to the corresponding author; to gain access, data requestors will need to sign a data access agreement.**

- In the online submission form, you indicated that : *due to the uniqueness of islet transplantation, the relatively low numbers receiving this treatment per year and the known location of the University of Alberta’s Clinical Islet transplant program, there is a risk that the data underlying the results may contain potentially identifying information. Therefore, the data underlying the results reported in this Article are not publicly available. De-identified individual participant data, as well as a data dictionary, can be made available to researchers who provide a methodologically sound proposal with ethics approval. Proposals should be directed to the corresponding author; to gain access, data requestors will need to sign a data access agreement.*
  - All PLOS journals now require all data underlying the findings described in their manuscript to be freely available to other researchers, either 1. In a public repository, 2. Within the manuscript itself, or 3. Uploaded as supplementary information.
    This policy applies to all data except where public deposition would breach compliance with the protocol approved by your research ethics board. If your data cannot be made publicly available for ethical or legal reasons (e.g., public availability would compromise patient privacy), please explain your reasons on resubmission and your exemption request will be escalated for approval.

**As described above, public availability would substantially compromise privacy and breach requirements of our HREB.**

- Please review your reference list to ensure that it is complete and correct. If you have cited papers that have been retracted, please include the rationale for doing so in the manuscript text, or remove these references and replace them with relevant current references. Any changes to the reference list should be mentioned in the rebuttal letter that accompanies your revised manuscript. If you need to cite a retracted article, indicate the article’s retracted status in the References list and also include a citation and full reference for the retraction notice.

**We believe we have reviewed cited literature and only include appropriate literature that have not been retracted.**
